# Neurons and Astrocytes in Ventrolateral Periaqueductal Gray Contribute to Restraint Water Immersion Stress-Induced Gastric Mucosal Damage via the ERK1/2 Signaling Pathway

**DOI:** 10.1093/ijnp/pyab028

**Published:** 2021-05-17

**Authors:** Wenting Gao, Zepeng Wang, Hui Wang, Huimin Li, Chenxu Huang, Yangyang Shen, Xiaoli Ma, Haiji Sun

**Affiliations:** 1 Key Laboratory of Animal Resistance Biology of Shandong Province, College of Life Science, Shandong Normal University, Jinan, China; 2 Research Center of Basic Medicine, Jinan Central Hospital, Cheeloo College of Medicine, Shandong University, Jinan, China

**Keywords:** Astrocyte, ERK1/2, restraint water-immersion stress (RWIS), neuron, ventrolateral periaqueductal gray (VLPAG)

## Abstract

**Background:**

The restraint water immersion stress (RWIS) model includes both psychological and physical stimulation, which may lead to gastrointestinal disorders and cause gastric mucosal damage. The ventrolateral periaqueductal gray (VLPAG) contributes to gastrointestinal function, but whether it is involved in RWIS-induced gastric mucosal damage has not yet been reported.

**Methods:**

The expression of glial fibrillary acidic protein, neuronal c-Fos, and phosphorylated extracellular signal regulated kinase 1/2 in the VLPAG after RWIS was assessed using western blotting and immunocytochemical staining methods. Lateral ventricle injection of astrocytic toxin L-a-aminoadipate and treatment with extracellular signal-regulated kinase (ERK)1/2 signaling pathway inhibitor PD98059 were further used to study protein expression and distribution in the VLPAG after RWIS.

**Results:**

The expression of c-Fos, glial fibrillary acidic protein, and phosphorylated extracellular signal regulated kinase 1/2 in the VLPAG significantly increased following RWIS and peaked at 1 hour after RWIS. Lateral ventricle injection of the astrocytic toxin L-a-aminoadipate significantly alleviated gastric mucosal injury and decreased the activation of neurons and astrocytes. Treatment with the ERK1/2 signaling pathway inhibitor PD98059 obviously suppressed gastric mucosal damage as well as the RWIS-induced activation of neurons and astrocytes in the VLPAG.

**Conclusions:**

These results suggested that activation of VLPAG neurons and astrocytes induced by RWIS through the ERK1/2 signaling pathway may play a critical role in RWIS-induced gastric mucosa damage.

**Significance Statement**

Although studies have implicated a role of ventrolateral periaqueductal gray (VLPAG) in gastrointestinal function, there have been no direct investigations on the function of VLPAG in restraint water immersion stress (RWIS)-induced gastric mucosal damage. This study is, to our knowledge, the first to investigate the interaction between astrocytes and neurons in the VLPAG during RWIS and the regulation of the ERK1/2 signaling pathway on the activity of astrocytes and neurons in the VLPAG following RWIS. The findings indicate that RWIS significantly increased p-ERK1/2, c-Fos, and GFAP expression in the VLPAG. Astrocytes activated by RWIS may directly impact VLPAG neuronal functioning, neuronal gene expression, and gastric dysfunction induced by RWIS. Our study provides new evidence for understanding the effects of RWIS-induced gastric mucosal damage on astrocytes and neurons in the VLPAG via the ERK1/2 signaling pathway.

## Introduction

The gastrointestinal (GI) tract is a primary target of stress, and it has been shown that stress is the most important etiologic factor in GI diseases. Serious stress has a great influence on GI secretion, motility, epithelial permeability, visceral sensitivity, microbiota, and inflammation (Konturek et al., 2008). Furthermore, these alterations might cause stress-related functional GI disorders such as irritable bowel syndrome and peptic ulcers. Stress-induced gastric mucosal injury (SGMI) is a typical example of stress-associated organ injury. RWIS mimics SGMI caused by trauma, surgery, or sepsis and has been widely accepted for studying SGMI (Spirt, 2004). A growing body of evidence has demonstrated that SGMI is closely related to gastric acid, pepsin secretion, gastric mucosal blood flow, prostaglandins, and gastric mucosal cell proliferation(Nishida et al., 1997; Li et al., 2006). However, the role of the central nuclei in regulating SGMI is still little known.

The gut-brain axis, the complex bidirectional communication between the GI tract and the central nervous system (CNS), plays a major role in numerous physiological and pathological conditions(Spencer and Hu, 2020). Brain-gut bidirectional communication comprises a complex reflex circuit. The central terminals of vagal afferent fibers innervate nucleus tractus solitarius (NTS) neurons and transduce and relay information about luminal distension and nutritional content from the GI tract. NTS neurons receive and integrate visceral sensory information and send axonal projections to adjacent motor nuclei, such as the dorsal motor nucleus of the vagus (DMV) as well as more distant higher CNS centers, including the hypothalamus and amygdala. The preganglionic parasympathetic motoneurons within the DMV innervate GI organs(Altschuler et al., 1993; Cottrell and Ferguson, 2004). The activity of DMV neurons is modulated by synaptic inputs from the NTS, the medullary raphe nuclei and area postrema within the brainstem as well as higher CNS nuclei, including the paraventricular nucleus (PVN) of the hypothalamus, central nucleus of the amygdala (CeA), and bed nucleus of the stria terminalis (Peters et al., 2008; Llewellyn-Smith et al., 2012). The complex (patho) physiology of brain-gut interactions may be involved in the mechanisms underlying stress-related gastric damage. In our previous study, the expression of glial fibrillary acidic protein (GFAP) and c-Fos in the PVN, DMV, NTS, locus coeruleus (LC), and CeA significantly increased following restraint water immersion stress (RWIS) (Zhang et al., 2009; Sun et al., 2018). Meanwhile, intracerebroventricular administration of astroglial toxin l-alpha-aminoadipate (L-AA) and c-Fos antisense oligodeoxy nucleotides decreased both RWIS-induced gastric mucosal damage and the activation of astrocytes and neurons induced by RWIS (Sun et al., 2016b; Fan et al., 2019). These findings revealed that neurons in these brain nuclei are involved in SGMI.

The VLPAG appears to be an integrative center that responds to hemorrhage, severe pain, trauma, and inescapable stress to produce behavioral and autonomic reactions that promote survival, wound healing, and passive protection from predator attack (Cavun and Millington, 2001; Vieira-Rasteli et al., 2018). Neuroanatomical tracing studies have shown that the VLPAG receives a dense innervation of endomorphinergic and catecholaminergic inputs from the NTS (Lv et al., 2010). Thus, parasympathetic sensory information from the GI tract can be relayed to the VLPAG. Together with the inputs from the hypothalamus and forebrain regions, including the CeA and the prefrontal and cingulate cortex, this allows the VLPAG to integrate and modulate autonomic and emotional information from all supraspinal levels (Parry et al., 2002; Butler et al., 2011). The VLPAG provides reciprocal connections back to many of these regions, including the CeA, hypothalamus, raphe magnus, LC, DMN, and NTS, which are involved in the regulation of GI functions. Meanwhile, our previous studies have shown that neuronal hyperactivity of the NTS, DMV, and LC may be one of the primary central mechanisms of gastric dysfunction induced by RWIS, while neuronal hyperactivity of the PVN and CeA may be one of the higher central mechanisms (He et al., 2018). It is reasonable to speculate that the VLPAG plays an important role in the mechanisms underlying the formation of stress-induced gastric damage.

Astrocytes are the most numerous neuroglial cells in the CNS. Astrocytes release several substances that act as gliotransmitters and influence astrocyte-neuron communication as well as neuronal activity and plasticity (Lavialle et al., 2011). Recent findings have suggested that astrocytes can directly sense and eventually change their morphology or function in response to stress (Young et al., 2008; Kirby et al., 2013). The expression of GFAP, as a marker of astrocytic activation, significantly increased in response to various physiological or noxious stimuli. Furthermore, some studies have demonstrated that the extracellular signal-regulated kinase (ERK) 1/2 pathway can influence GFAP expression and is involved in regulating astrocyte activation (Yang et al., 2016). The ERK1/2 pathway is activated in astrocytes under ischemia and might protect astrocytes from ischemic injury (Hao et al., 2011). Meanwhile, phosphorylation of ERK1/2 is one of the major pathways for the induction of c-Fos. Morphine withdrawal–induced overexpression of c-Fos has been colocalized with the phosphorylation of ERK in the VLPAG (Jiang et al., 2002). So far, these results lead us to hypothesize that astrocytes and neurons are involved in SGMI via the ERK1/2 pathway.

In this study, to explore whether neurons and astrocytes within the VLPAG are involved in SGMI, immunohistochemistry and western blotting were used to examine the expression of Fos, GFAP, and p-ERK1/2. Furthermore, we microinjected L-AA to investigate whether astrocytes participate in RWIS-induced gastric mucosa damage by regulating neuronal activity. The ERK1/2 signaling pathway inhibitor PD98059 was utilized to identify its role in the regulation of neurons and astrocytes during RWIS.

## Materials and Methods

### Animals

Male Wistar rats weighing approximately 220–250 g were purchased from the Experimental Animal Center of Shandong University. The animals were housed in a controlled environment (22°C ± 2°C) on a 12-hour-light/-dark cycle and had free access to water and standard rat fodder. All rats were deprived of food, but not water, overnight before RWIS. All experiments were conducted according to the guidelines of the International Association for the Study of Pain (Zimmermann, 1986) and approved by the Experimental Animal Ethical Association of Shandong Normal University.

### Experimental Design

The animals were randomly divided into 5 groups in accordance with the duration of RWIS (n = 6 in each group/treatment): control group, and experimental groups treated with RWIS for 0.5 hour, 1 hour, 3 hours, and 5 hours. The rats in the experimental groups were anesthetized using soflurane and fixed on a board. After the rats were conscious, they were vertically immersed in water (21°C ± 1°C) to the level of the xiphoid for 0.5 hour, 1 hour, 3 hours, and 5 hours. To eliminate the effect of circadian rhythm on the rats, the experiment was conducted between 8:00 am and 1:00 pm.

### Administration of Drugs

The rats were anesthetized with isoflurane and fixed in a stereotaxic apparatus. Stereotaxic coordinates were: 0.8 mm posterior, 1.8 mm right lateral to bregma, and 3.8 mm ventral from bregma according to Paxinos and Watson’s brain atlas. The rats were randomly divided into 2 groups: the saline group and the L-AA group. The astroglial toxin L-AA (dissolved 100 nmol in 10 μL physicological saline, Sigma, St. Louis, MO) or physiological saline (control, 10 µL physiological saline) was then injected into the lateral ventricle. The solutions were injected into the lateral ventricles at constant speed for 10 minutes. After the injection, the rats were subjected to RWIS for 1 hour and were killed immediately after RWIS, and the expression of c-Fos and GFAP in the VLPAG was measured with immunohistochemical staining.

PD98059 is an inhibitor of the MAPKK family members MEK1/2. To examine whether the MAPKK signaling pathway participates in the damage to gastric mucosal induced by RWIS, the rats were randomly divided into 2 groups: the control group was injected with 10 µL normal saline into the lateral ventricle, and the PD98059 group was injected with PD98059 (0.2 mg dissolved in 10 µL dimethyl sulfoxide [DMSO]) into the lateral ventricle. For each injection, the solution was slowly and steadily injected for 10 minutes, and then the rats were treated with RWIS for 1 hour. The expression of GFAP, c-Fos, and p-ERK1/2 in the VLPAG was subsequently detected with immunohistochemistry or western blotting.

### Immunohistochemistry

Rats were perfused with 0.01 M phosphate-buffered saline (PBS, pH 7.4) followed by 250 mL freshly prepared 4% paraformaldehyde in 0.1 M phosphate buffer (4°C). The brains were removed immediately, postfixed for 4 hours, and dehydrated overnight in 20% sucrose in 0.1 M phosphate buffer saline (PBS) at 4°C. Thirty-micrometer successive coronal sections containing the VLPAG were cut and then collected into 0.01 M PBS. Endogenous active enzymes were blocked with 2% goat serum in 0.01 M PBS containing 0.3% Triton X-100 for 1 hour at room temperature. The GFAP-immunoreactive (GFAP-IR), c-Fos-immunoreactive (c-Fos-IR), and p-ERK1/2-immunoreactive (p-ERK 1/2-IR) sections were incubated overnight at 4°C with rabbit anti-GFAP 1:300 (Bioss Antibodies, Beijing, China), rabbit anti-c-Fos (1:500; Santa Cruz Biotechnology, Dallas, TX, USA), and rabbit anti-p-ERK1/2 (1:1000; Cell Signaling Technology, Danvers, MA, USA). Subsequently, the sections were incubated with biotinylated goat anti-rabbit IgG (Zymed Laboratories, San Francisco, CA, USA) for 1 hour at room temperature. The specificities of the staining were tested on the sections in the control rats by omitting the primary antibodies.

### Western Blotting

After RWIS, we collected the VLPAG and extracted the proteins. Protein extracts were separated by sodium dodecyl sulfate-polyacrylamide gel Electrophoresis followed by transfer onto Polyvinylidene Fluoride membranes. Subsequently, the membrane was blocked with blocking buffer (5% non-fat dry milk and 0.1% Tween-20 contained in 0.01 M PBS) at 4°C overnight. The following primary antibodies were added: rabbit anti-GFAP (1:500), rabbit anti-GAPDH (1:500), and rabbit anti-p-ERK1/2 (1:1000). After rinsing in Tris Buffered saline Tween buffer 3 times, the membrane was incubated with secondary antibodies for 1 hour at RT. Enhanced chemiluminescence staining was used, and the membrane was scanned by image analysis software. Quantity One was used to measure the optical density (Fan et al., 2018).

### Evaluation of Gastric Mucosal Damage

After the rats were killed, their stomachs were removed and injected with 10 mL of 4% paraformaldehyde solution. The stomachs were incised along the greater curvature of the stomach and then rinsed with normal saline. Gastric mucosal lesions were identified with a magnifying lens. The erosion index (EI) was measured as follows: length ≤1 mm was 1, 1 mm < the length ≤2 mm was 2, and the others were deduced in turn. The score was multiplied by 2 when the width of the lesion was larger than 1 mm. The cumulative scores of all lesions in a rat served as the EI of the rat (Guth, 1992).

### Statistical Analysis

All of the data were analyzed with SPSS 16.0 software. Statistical analysis was conducted with an independent-samples *t* test or 1-way ANOVA with the least-significant difference test. The results are reported as the mean values ± SEM. *P* < .05 was considered a significant difference. *P* < .01 was considered a very significant difference.

## Results

### Effects of RWIS on Gastric Mucosal Damage

**Figure 1.**
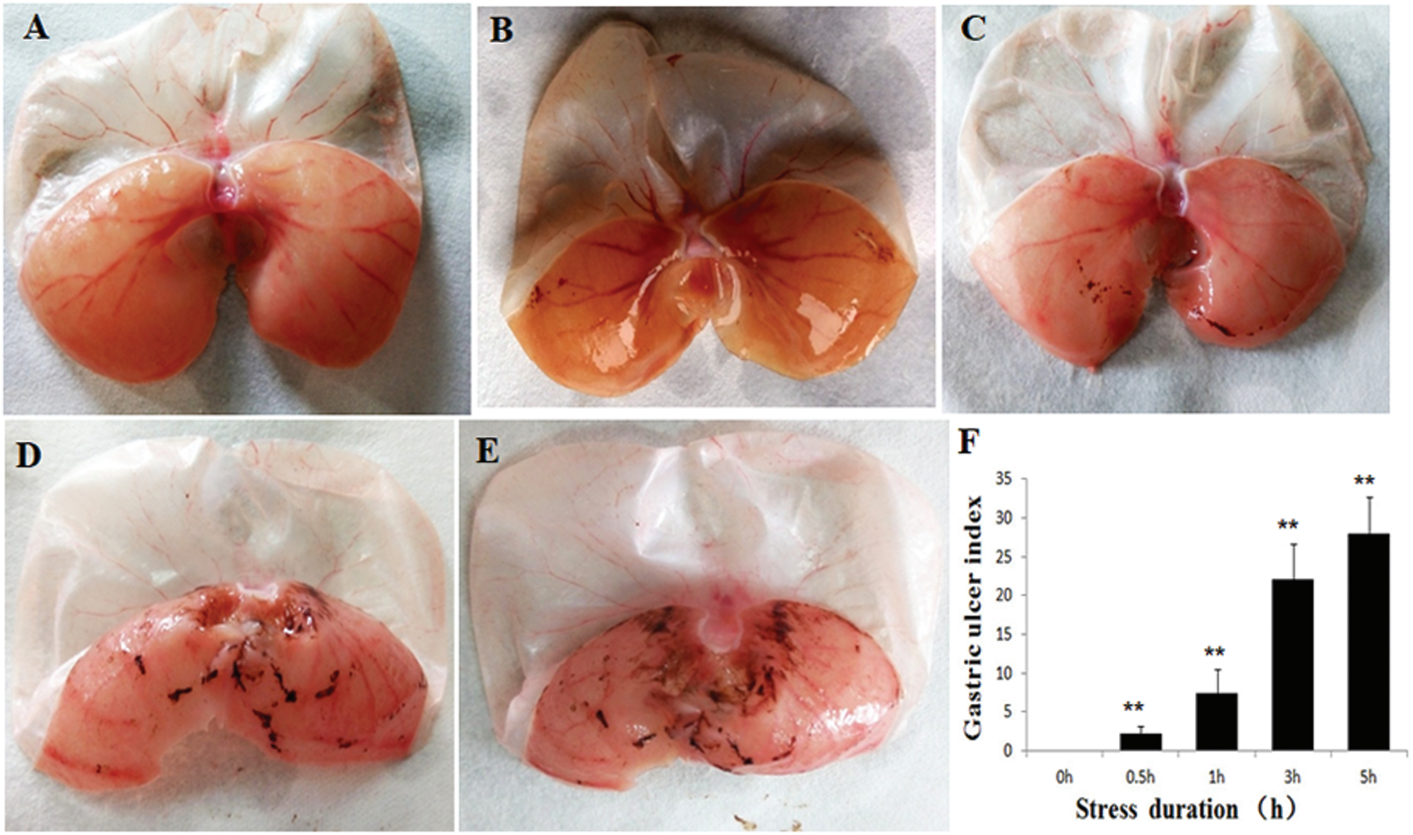
Effects of restraint water immersion stress (RWIS) on gastric mucosal damage. (A–E) RWIS 0 hours (control), 0.5 hour, 1 hour, 3 hours, and 5 hours. (F) Quantification of the erosion index (EI). All data are expressed as the mean ± SEM (n = 6). **P < .01 vs control group.

Compared with the control group, the index of gastric mucosal injury increased significantly at different periods of RWIS (Figure 1A–E). The gastric mucosal surface in the control group was intact and not damaged (Figure 1A); however, scattered spots or lineal hemorrhage lesions (supplemental Figure 1) were observed in the mucosa in the 0.5-hour, 1-hour, 3-hour, and 5-hour groups under continuous stress, indicating that there is a significant effect of RWIS on gastric mucosal damage (Figure 1F).

### C-Fos and GFAP Expression in the VLPAG of RWIS Rats

**Figure 2.**
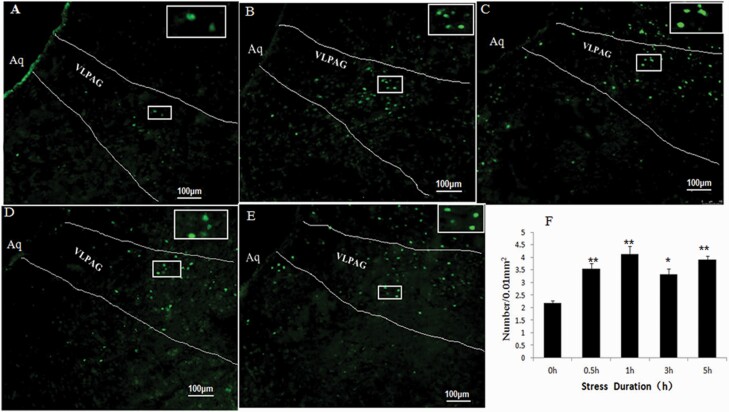
c-Fos-positive neurons in the ventrolateral periaqueductal gray (VLPAG) induced by RWIS. (A–E) c-Fos-IR neurons in the VLPAG induced by RWIS at 0 hours, 0.5 hour, 1 hour, 3 hours, and 5 hours of RWIS (100×). Fos-IR neurons in a rectangle were magnified to a higher magnification (400×). (F) The number of Fos-IR neurons in the VLPAG was quantified. ** *P* < .01, *P < .05, compared with control group.

**Figure 3.**
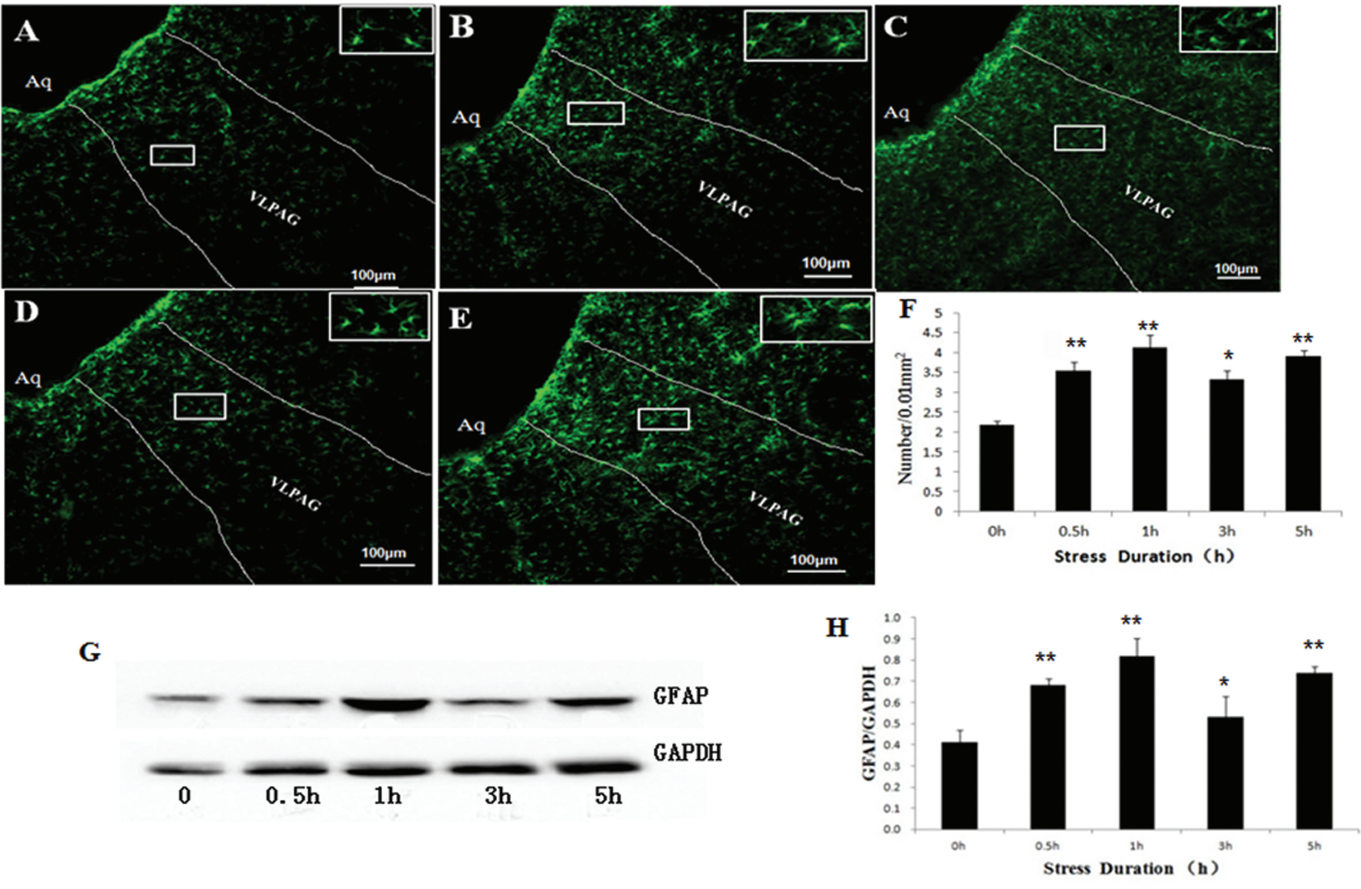
The glial fibrillary acidic protein (GFAP)-positive astrocytes in the VLPAG induced by RWIS. (A-E) GFAP-IR astrocytes in the VLPAG induced by RWIS at 0 hours, 0.5 hour, 1 hour, 3 hours, and 5 hours of RWIS (100×). GFAP-IR astrocytes in a rectangle were magnified to a higher magnification (400×). (F) The number of GFAP-IR astrocytes in the VLPAG was quantified. (): Western blot of GFAP protein induced by RWIS. (H) Quantification of the relative average grayscale value of GFAP/GAPDH (n = 6). **P < .01, *P < .05 compared with the control group.

To explore the effect of RWIS on neuronal activity in the VLPAG, the expression of the c-Fos protein, a marker of neuronal activity, was assessed using immunohistochemical staining. Compared with the control group, c-Fos expression was significantly increased at 1 hour, 3 hours, and 5 hours after RWIS, with its peak at 3 hours after RWIS (Figure 2A–F). These results indicated that neurons in the VLPAG are involved in the RWIS process. To investigate whether astrocytes in the VLPAG are involved in RWIS, we detected the number of GFAP-positive astrocytes and amount of GFAP protein expression in the VLPAG by immunohistochemical staining and western blotting. During RWIS treatment (0.5 hour, 1 hour, 3 hours, and 5 hours), the number of GFAP-positive astrocytes in the VLPAG was significantly increased compared with that in the control group (Figure 3A–F). The number of GFAP-positive astrocytes peaked at 1 hour after RWIS and then decreased gradually at 3 hours but remained higher than that in the 0-hour group (*P* < .0l) and reached a second peak at 5 hours. The western-blotting results were consistent with the immunohistochemical staining results (Figure 3G–H). These results indicated that astrocytes in the VLPAG may be involved in the RWIS process.

### Effects of L-AA on Gastric Mucosal Damage, c-Fos, and GFAP Expression in the VLPAG Induced by RWIS

**Figure 4.**
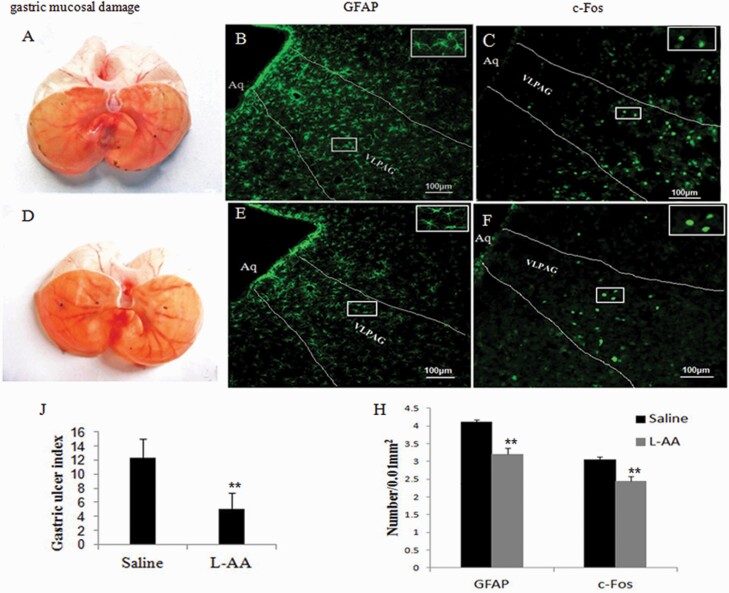
Effects of l-alpha-aminoadipate (L-AA) on gastric mucosal damage, c-Fos and GFAP expression induced by RWIS. (A) Gastric mucosal damage in RWIS rats treated with saline. (B–C) GFAP and c-Fos expression in RWIS rats treated with saline (100×); scale bar, 100 μm. (D) Gastric mucosal damage in RWIS rats treated with L-AA. (E–F) GFAP and c-Fos expression in RWIS rats treated with L-AA (100×); scale bar, 100 μm. (J) Quantification of erosion index (EI). All data are expressed as the mean ± SEM (n = 6). (H) Quantification of GFAP-IR in astrocytes and Fos-IR in neurons (n = 6). **P < .01 vs control group.

To explore the interaction between astrocytes and neurons during RWIS, the astrocytic toxin L-AA was used. RWIS rats treated with saline demonstrated significant gastric mucosal damage (Figure 4A); however, L-AA treatment significantly reduced gastric mucosal damage (Figure 4D and J) and significantly decreased RWIS-induced GFAP expression (Figure 4B, E, and H). In addition, RWIS-induced c-Fos expression in the VLPAG was also obviously reduced compared with that in the saline control group (Figure 4C, F, and H). These results demonstrated that the interaction between neurons and astrocytes in the VLPAG may participate in RWIS-induced gastric mucosa damage.

### Increased Expression of p-ERK1/2 in the VLPAG During RWIS

**Figure 5.**
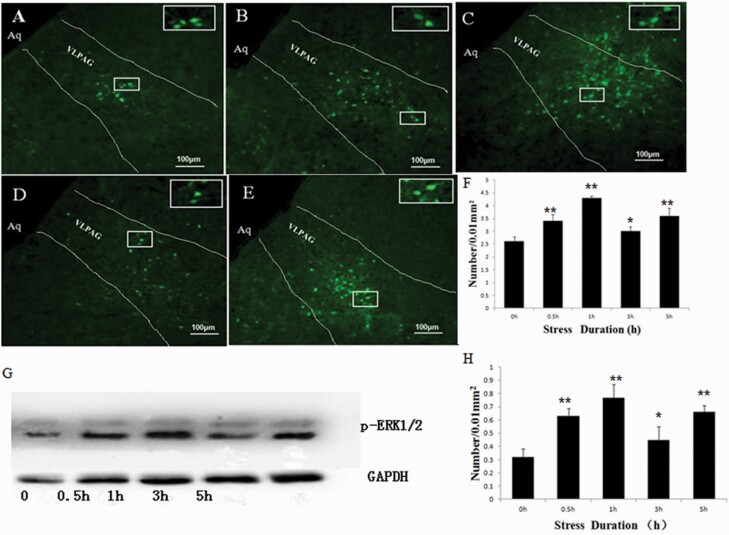
Immunohistochemical staining and western blotting for p-ERK1/2 protein induced by RWIS. (A–E) p-ERK1/2-positive neurons in the VLPAG induced by RWIS at 0 hours, 0.5 hour, 1 hour, 3 hours, and 5 hours (100×). p-ERK1/2 in a rectangle was magnified to a higher magnification (400×). (F) Quantification of positive neurons. (G) Western blotting of p-ERK1/2 protein induced by RWIS. (H) Quantification of the relative average grayscale value of p-ERK1/2/GAPDH (n = 6). **P < .01, *P < .05 vs control group.

To determine whether the ERK1/2 signaling pathway was involved in RWIS, the expression of p-ERK1/2 was examined by immunohistochemical staining and western blotting. Immunostaining for p-ERK1/2 was used to examine the distribution of RWIS-induced ERK activation following RWIS treatment. p-ERK1/2-positive neurons were observed in the VLPAG (Figure 5A–E). The time course of the number of p-ERK1/2-positive neurons after RWIS is illustrated in Figure 5F. Compared with the 0-hour group, the number of p-ERK1/2-positive neurons significantly increased at 0.5 hour, reached a peak at 1 hour, decreased at 3 hours, and then reached a second peak at 5 hours after RWIS treatment. Western immunoblots for p-ERK1/2 protein expression are depicted in Figure 5G and H. A repeated-measure ANOVA confirmed that p-ERK1/2 expression in the VLPAG was significantly upregulated following RWIS. The temporal pattern of p-ERK1/2 expression was consistent with the immunohistochemistry results. These results suggest that ERK1/2 signaling pathway participates in RWIS.

### Effects of PD98059 on the Gastric Mucosal Damage, p-ERK1/2, c-Fos, and GFAP Expression Induced by RWIS

**Figure 6.**
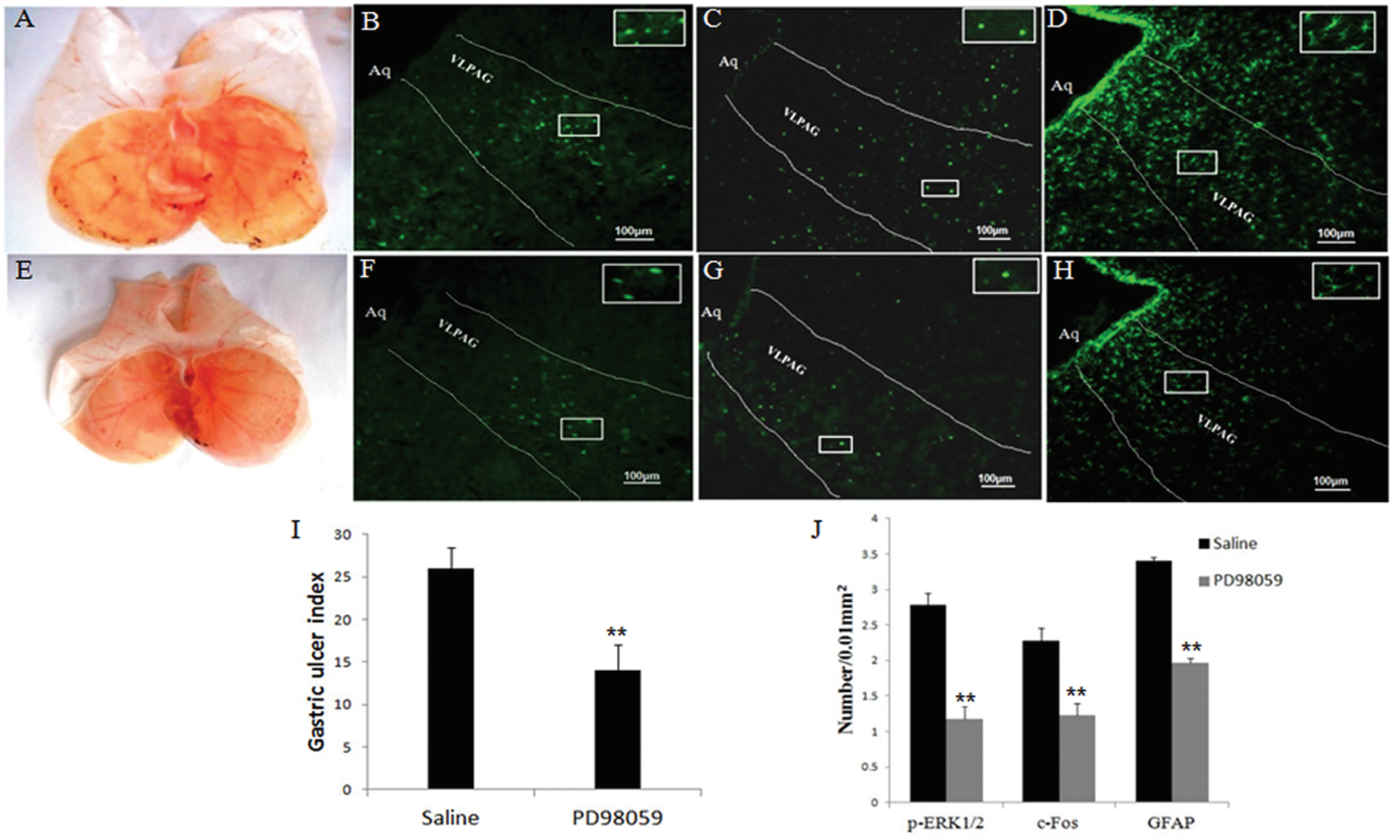
Effects of PD98059 on the gastric mucosal damage, p-ERK1/2, c-Fos, and GFAP expression induced by RWIS. (A) Gastric mucosal damage in RWIS rats treated with saline. B-D: p-ERK1/2, c-Fos, and GFAP expression in RWIS rats treated with saline (100×); scale bar, 100 μm. (E) Gastric mucosal damage in RWIS rats treated with PD98059. (E–H) p-ERK1/2, GFAP, and c-Fos expression in RWIS rats treated with PD98059 (100×); scale bar, 100 μm. (I) Quantification of erosion index (EI). All data are expressed as the mean ± SEM (n = 6). (J) Quantification of p-ERK1/2 and GFAP-IR in astrocytes.

To explore the role of the ERK1/2 signaling pathway in the process of RWIS, the upstream inhibitor of ERK phosphorylation, PD98059, was microinjected into the lateral ventricle. Intracerebroventricular injection of PD98059 significantly alleviated RWIS-induced gastric mucosal damage (Figure 6A, E, and I). Meanwhile, injection of PD98059 had an obvious inhibitory effect on RWIS-induced p-ERK1/2-positive neurons (Figure 6B, F, and J). Moreover, both the number of c-Fos-positive neurons (Figure 6C, G, and J) and the number of GFAP-positive astrocytes significantly decreased following treatment with PD98059. These results indicate that the ERK1/2 signaling pathway may regulate the activity of neurons and astrocytes in the VLPAG and is involved in RWIS-induced gastric mucosa damage.

## Discussion

Data obtained from the present study showed that RWIS strongly induced astrocytic and neuronal activation in the VLPAG and increased gastric mucosal damage. In addition, intracerebroventricular administration of the astroglial toxin L-AA reduced RWIS-induced gastric mucosal damage and suppressed the activation of both astrocytes and neurons induced by RWIS. These results demonstrated that the astrocytes in the VLPAG may regulate neuronal activity and participate in RWIS-induced gastric mucosa damage. Moreover, in the present study, RWIS caused an increase in p-ERK1/2 activation in the VLPAG, and the ERK1/2-specific inhibitor PD98059 attenuated the development of gastric mucosal damage induced by RWIS and decreased astrocytic and neuronal activity, suggesting that the ERK1/2 signaling pathway may mediate the activation of the neuron-astrocyte network in the VLPAG following RWIS.

The PAG refers to the region of the midbrain that surrounds the cerebral aqueduct and consists of 4 longitudinal columns along the rostrocaudal region located dorsomedial, dorsolateral, lateral, and ventrolateral to the cerebral aqueduct. Much evidence suggests that the midbrain PAG plays a pivotal role in mediating animal responses to threatening, stressful, or painful stimuli. The VLPAG appears to be an integrative center that responds to hemorrhage, severe pain, trauma, and inescapable stress to produce behavioral and autonomic reactions that promote survival, wound healing, and passive protection from predator attack(Parsons, 2008; Taylor et al., 2019). Following noxious stimulation of hindlimb muscle, knee joint, or peritoneal nociceptors, there was a significant increase in the numbers of Fos-IR cells in the VLPAG (Parry et al., 2002). Bilateral injection of lidocaine into the caudal VLPAG delayed the onset and significantly reduced the magnitude of the hypotension produced by hemorrhage stress, which suggests that the VLPAG plays an important role in the response to hemorrhage stress(Cavun and Millington, 2001). Irreversible chemical lesions of the VLPAG promoted a reduction in the immobility stress response and blocked defensive antinociception, indicating that the VLPAG is a key region for regulating immobility stress and is essential for defensive antinociception in guinea pigs(Leite-Panissi et al., 2003). Neuroanatomical tracing studies have shown that the VLPAG receives dense innervation of endomorphinergic and catecholaminergic inputs from the NTS. Thus, parasympathetic sensory information from the GI tract can be relayed to the VLPAG (Farkas et al., 1997; Viltart et al., 2006). Together with the inputs from the hypothalamus and forebrain regions, including the CeA and the prefrontal and cingulate cortexa, this allows the VLPAG to integrate and modulate autonomic and emotional information from all supraspinal levels. The VLPAG provides reciprocal connections back to many of these regions, including the central nucleus of the CeA, the hypothalamus, the raphe magnus, the LC, DMN, and NTS, which are involved in the regulation of GI functions. Meanwhile, our previous studies have shown that neuronal hyperactivity of the NTS, DMV, and LC may be one of the primary central mechanisms of gastric dysfunctions induced by RWIS (Sun et al., 2016a; Fan et al., 2019), while neuronal hyperactivity of the PVN and CeA may be one of the higher central mechanisms(Sun et al., 2016b; He et al., 2018). It is reasonable to speculate, therefore, that the VLPAG plays an important role in the mechanisms underlying the formation of SGMI. It is known that c-Fos expression is an accepted marker for neural activation. The present study utilizing c-Fos immunocytochemistry demonstrated that the number of Fos-IR cells in the VLPAG after RWIS significantly increased compared with that in the control group, which suggests that neurons in the VLPAG were excited during RWIS. Therefore, it is possible that neurons in the VLPAG were activated and involved in gastric dysfunction induced by RWIS, leading to stress-induced gastric mucosa damage.

Abundant recent evidence has demonstrated that glial cells, particularly astrocytes, envelop neuronal synapses and participate in the regulation of the functional activity of the nervous system via the release of synaptically effective mediators, a process called gliotransmission(Bains and Oliet, 2007; Halassa et al., 2007). Evidence has now shown that chronic and acute stress can induce alterations in the morphology and function of astrocytes, which may impair brain function and ultimately be involved in the pathophysiology of stress-related disorders(Feng et al., 2010). It has been reported that chronic restraint stress induces a significant decrease in GFAP protein levels in the VLPAG (Imbe et al., 2012). In addition, peripheral nerve injury acute stress significantly increased the number of GFAP-IR cells in the VLPAG (Mor et al., 2010). However, no study has examined GFAP protein levels in the VLPAG after RWIS. In the current study, we found that RWIS significantly increased GFAP expression in the VLPAG compared with the control group. It is well known that the upregulation of GFAP levels is associated with functional changes in astrocytes, which, in turn, affect neuronal function(Gómez-Galán et al., 2013). Therefore, these findings suggest that RWIS-induced dysfunction of astrocytes in the VLPAG may be involved in the development of abnormal gastric function and contribute to gastric mucosal damage after RWIS.

In recent years, considerable evidence has demonstrated the existence of reciprocal communication between astrocytes and neuronal cells. Astrocytes sense the same synaptic inputs as neurons and elevate intracellular calcium. Astrocyte calcium signaling triggers the release of gliotransmitters such as glutamate, ATP, and D-serine, which modulate presynaptic neurotransmitter release and affect neuronal activity and function(Garcia-Segura et al., 2008; Bernardinelli et al., 2011). Thus, impairment of astrocytes induced by chronic restraint stress is considered to affect neuronal function in the PAG. Recent studies have reported that vPAG a1ARs drive glutamatergic excitation of vPAGDA neurons, are located on astrocytes, and promote wakefulness (Porter-Stransky et al., 2019). Meanwhile, recent data have shown that chronic opioid exposure induces astrocyte activation and proinflammatory mediator expression in the PAG associated with the complex syndrome of opioid dependence. Specifically, increased TNFα on astrocytes activated by opioids directly impacts PAG neuronal functioning (Ouyang et al., 2012). Thus, it is possible that activated astrocytes and neurons interact to contribute to gastric mucosal damage after RWIS. To investigate the relationship between neurons and astrocytes in the VLPAG, we used an intracerebroventricular injection of L-AA to inhibit activated astrocytes in the VLPAG induced by RWIS. L-AA, a gliotoxin specific for astrocytes, enters cells via sodium (Na)-dependent transporter and blocks essential cellular functions involving glutamate, including protein synthesis and energetic metabolism, thereby inducing glial death (Brown and Kretzschmar, 1998). Inhibition of astrocyte activation by L-AA has been previously reported to reduce pathological pain responses (Jiang et al., 2016) and attenuate the peripheral nerve injury–induced neuropathic pain (Nam et al., 2016). Our results showed that the injection of L-AA in rats significantly alleviated the extent of gastric mucosal damage. Moreover, the astroglial toxin L-AA not only decreased GFAP expression but also inhibited c-Fos expression, which suggests that astrocytes in the VLPAG were activated by RWIS and then modulated the activity of neurons via the neuron-astrocyte network. Previous studies have demonstrated that activation of astrocytes induces the synthesis and release of substances (e.g., cytokines and glutamates) capable of modulating the functions of surrounding cells, including neurons (Watkins et al., 2007). Accordingly, in the present study, activated astrocytes may produce gliotransmitters (i.e., ATP) and influence neuronal activity in the VLPAG, which contributes to RWIS-induced gastric mucosal damage.

ERK1/2 is a family of serine/threonine protein kinases activated by extensive stimuli, involved in stress reactions, and utilized as a marker for the reaction of neurons to stress (Shen et al., 2004; Kwon et al., 2006). Immobilization stress increased pERK-IR cells in the VLPAG, and chewing downregulated this increase (Yamada et al., 2015). In the present study, p-ERK1/2 expression in the VLPAG was significantly increased following RWIS; moreover, pretreatment with PD98059 attenuated RWIS-induced gastric mucosa damage. These results suggested that the ERK1/2 signaling pathway was activated during RWIS and was involved in the process of RWIS. Much of the previous research demonstrated that phosphorylation of ERK1/2 is one of the major pathways for the induction of c-Fos (Radwanska et al., 2005; Núñez et al., 2008). Morphine withdrawal-induced overexpression of c-Fos colocalizes with the phosphorylation of ERK in the VLPAG (Hao et al., 2011). In the current study, we observed an increase in p-ERK and c-Fos expression in the VLPAG following RWIS. Both p-ERK1/2 and c-Fos expression significantly increased at 0.5 hour, peaked at 1 hour, and started gradually decreasing at 3 hours after RWIS, demonstrating a similar temporal pattern of the expression of the 2 proteins. Furthermore, treatment with PD98059 not only prevented p-ERK1/2 but also blocked c-Fos expression. Therefore, it is possible that the ERK1/2 signaling pathway modulated c-Fos expression and influenced neuronal activity in the VLPAG during RWIS and then participated in RWIS-induced gastric mucosa damage.

Several lines of evidence have revealed that the ERK1/2 signaling pathway can be activated in response to growth factors, oxidative stress, and ischemic injury. These stimulating factors display different effects by either activating or inhibiting the phosphorylation of ERK1/2, which further changes the processes of astrocyte proliferation, differentiation, and apoptosis (Boulos et al., 2007; Hsieh et al., 2010). The ERK1/2 pathway is activated in astrocytes under ischemia and might protect astrocytes from ischemic injury (Jiang et al., 2002). The results of the present study showed that PD98059, an ERK inhibitor, significantly inhibited the activation of the ERK1/2 signaling pathway induced by RWIS, which led to decreased astrocytic GFAP expression in the VLPAG. In addition, the administration of PD98059 significantly attenuated RWIS-induced gastric mucosal damage. Therefore, it is possible that the ERK1/2 signaling pathway plays an important role in regulating the activation of the neuron-astrocyte network under RWIS conditions and promotes damage to the gastric mucosa. Here, we mainly used immunohistochemistry quantifications to detect the expression of c-Fos in the VLPAG during the RWIS and determine whether the VLPAG is involved in RWIS-induced mucosal damage. To verify the specificity of the immunohistochemistry method, we tested c-Fos expression in the ventrolateral medulla during the RWIS. The results showed that the expression of c-Fos in the ventrolateral medulla had no significant difference between the control rats and RWIS rats (supplementary Figure 2). These results at least in part indicated that the VLPAG is specific to the involvement of stress-induced gastric mucosal damage. Furthermore, dual-immunohistochemistry should be employed to explore which types of neurons in the VLPAG are activated and involved in the process of RWIS.

In summary, we have demonstrated that RWIS significantly increased p-ERK1/2, c-Fos, and GFAP expression in the VLPAG. Astrocytes activated by RWIS may directly impact VLPAG neuronal functioning, neuronal gene expression, and gastric dysfunction induced by RWIS. Furthermore, the activation of the neuron–astrocyte signaling network in the VLPAG through the ERK1/2 signaling pathway may play a role in RWIS-induced gastric mucosa damage. Our previous study has shown that many central nuclei, such as the DMV, NTS, PVN, and CeA, are activated and involved in the formation of stress-induced gastric mucosal damage. However, the interaction of these nuclei in the RWIS and the effect on gastric mucosal damage are rarely studied. Meanwhile, many neurotransmitters or neuromodulators, such as nitric oxide, vasoactive intestinal peptide, substance P, acetylcholine, catecholamine, glutamate, γ-aminobutyric acid, and oxytocin, functionally or anatomically linked to the CNS and may participate in the regulation of RWIS. Therefore, future studies should investigate the specific role of these nuclei and the neurotransmitters or neuromodulators, which may lead to a better understanding of the pathophysiology of stress-induced gastric mucosal damage.

## Supplementary Material

pyab028_suppl_Supplementary_Figure_S1

pyab028_suppl_Supplementary_Figure_S2
