# Supplementary figures and images for: Neurons and Astrocytes in Ventrolateral Periaqueductal Gray Contribute to Restraint Water Immersion Stress-Induced Gastric Mucosal Damage via the ERK1/2 Signaling Pathway
Source: Int J Neuropsychopharmacol. 2021 May 17;24(8):666–76. doi: 10.1093/ijnp/pyab028 (PMC8378083; doi:10.1093/ijnp/pyab028)

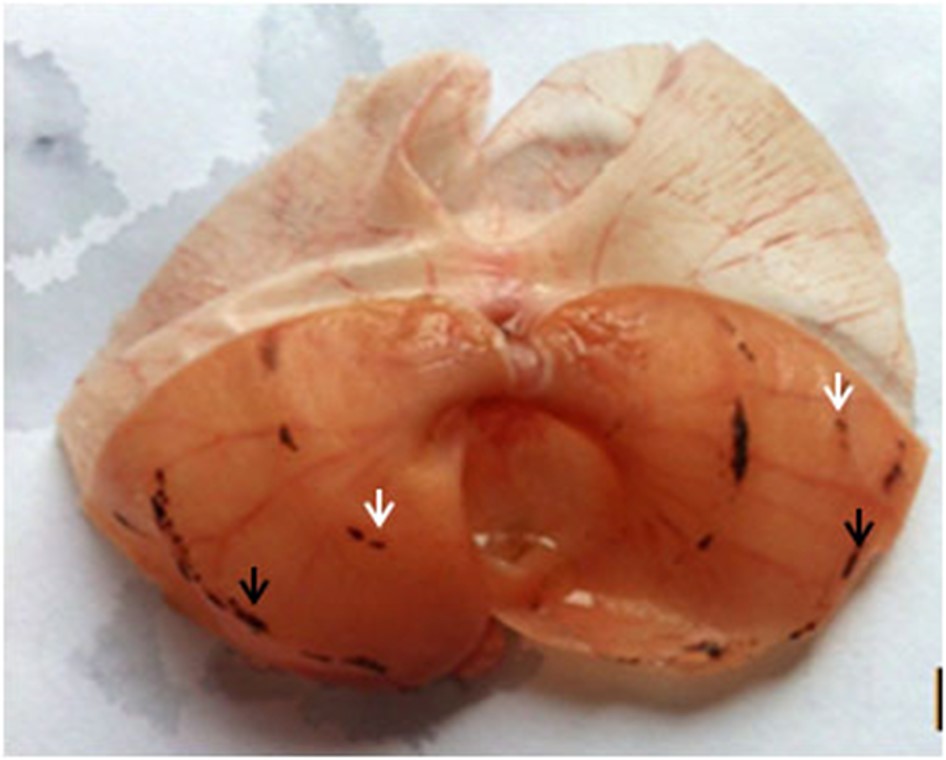

Supplement: pyab028_suppl_Supplementary_Figure_S1 [file pyab028_suppl_supplementary_figure_s1.jpeg]

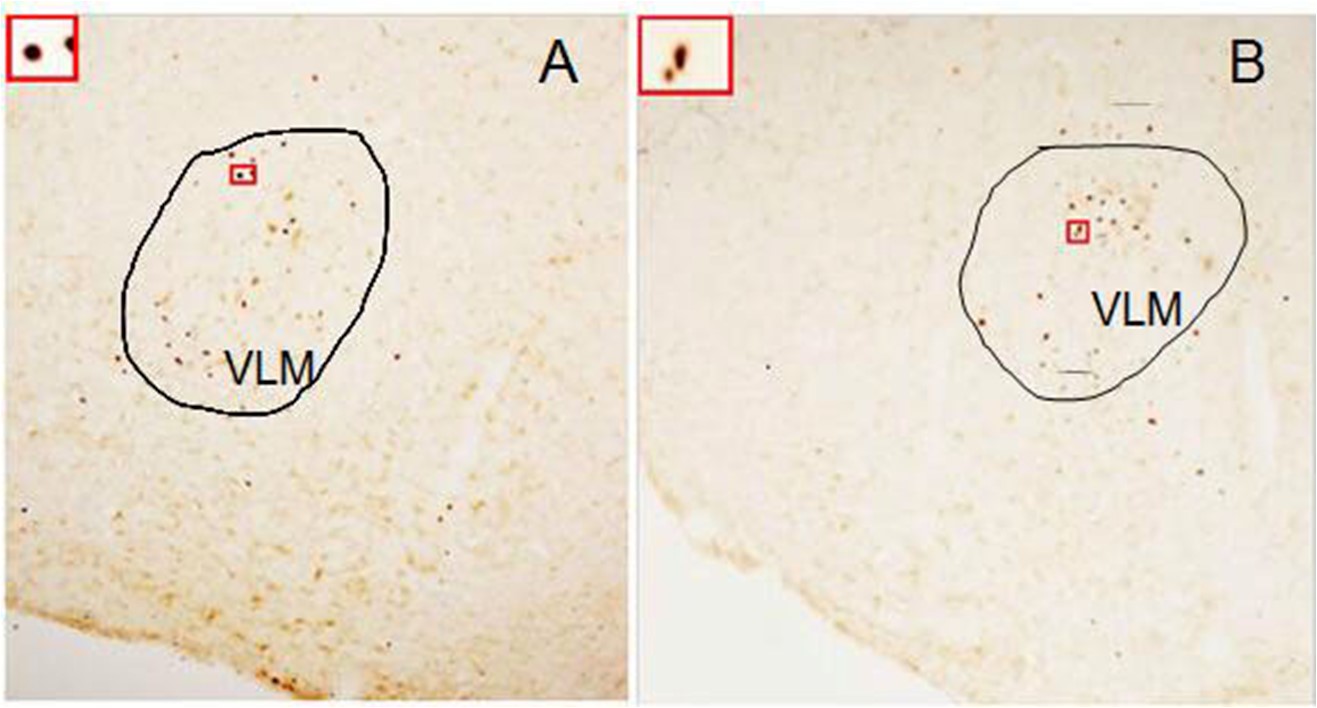

Supplement: pyab028_suppl_Supplementary_Figure_S2 [file pyab028_suppl_supplementary_figure_s2.jpeg]
